# The effect of marital satisfaction on the self-assessed depression of husbands and wives: investigating the moderating effects of the number of children and neurotic personality

**DOI:** 10.1186/s40359-023-01200-8

**Published:** 2023-05-17

**Authors:** Le Yang, Ziqi Yang, Jingjing Yang

**Affiliations:** 1 School of Public Health, Chongqing Medical University, Chongqing, China; 2 Research Center for Medicine and Social Development, Chongqing Medical University, Chongqing, 400016 China

**Keywords:** Self-rated depression, Actor–partner interdependence moderation model, Couple, Marriage, Family

## Abstract

**Background:**

Based on the family system theory, there is an interactive relationship in the family, especially the cognitive style and emotional changes of the husband and wife will affect the behavior, cognition and emotion of the partner. Data about the effects of marital relationships on mental health are often paired. Scholars study the effect of individual independent variables on the dependent variables and the effect of spouse independent variables on the dependent variables to explore the actor and partner effect in marital relationships.

**Methods:**

This study used the China Family Panel Studies (CFPS) 2018 dataset to collect paired data on the marital satisfaction and self-rated mental health of 9,560 couples. The Actor–Partner Interdependence Moderation Model (APIMoM) was used to analyze whether moderator variables affect the direction and strength of the effect of marital satisfaction on self-rated depression. In the robustness test part, the robustness of the APIMoM model was tested by reanalyzing the independent variables using two kinds of  binary codes respectively, and the results showed that the models were robust.

**Results:**

Individuals’ marital satisfaction was significantly negatively correlated with their own depression level and with that of their spouse. The number of family members had a positive moderating effect on the results of the wife’s partner effect. Couples who lived in the environment with more family members had lower depression scores. Couples who have more children have higher depression scores. The number of children has a negative moderating effect on the results of partner effect of husbands and wives. The wife’s neurotic personality score has a negative moderating effect on the wife’s actor effect.

**Conclusions:**

In terms of measures to prevent depression, women’s mental health should be given more priority than men’s. Living in a larger family with more children is beneficial for couples’ mental health. Efforts to prevent depression in couples should take into account the neurotic character of the members, especially the wife, and design special treatment and preventive measures accordingly. These findings highlight that binary dynamics should be considered in exploring what factors influence the mental health of married couples.

## Background

Marital disharmony is regarded as an essential risk factor for depressive symptoms in couples in the Marital Discord Model of Depression (MDMD) [1]. In a meta-analysis of 26 cross-sectional studies, the magnitude of the effect of marital satisfaction on depression was − 0.42 for women and − 0.37 for men [2]. In addition, prospective studies have shown that dissatisfaction with marital relationships predicts subsequent depressive symptoms, and improving marital satisfaction and sexual satisfaction can prevent the development of adverse emotions such as depression and anxiety [3, 4]. A study by Whisman [5] found that people’s depression and anxiety levels were significantly correlated with their marital satisfaction; however, the depression effect was significantly stronger than the anxiety effect. Moreover, the depression levels of both members of a couple were found to have a significant interaction in this study, but the same effect was not observed for their anxiety levels. In another study, marital satisfaction amplified the negative relationship between disability and life satisfaction as a moderator [6]. Most previous studies on the relationship between marital satisfaction and depressive symptoms have focused on the actor effect; nonetheless, the partner effect has also been increasingly investigated [7]. The theoretical biologist Von Bertalanffy [8], who founded the system theory, believed that the system refers to the collection of things that interact with each other and depend on each other in a regular way, which is an orderly whole. Generally speaking, there are at least three conditions for forming a system: (1) the system is composed of many different components; (2) The components are not isolated, but interconnected and interacted with each other; (3) The system has independent and specific functions. An ecosystem refers to an ecological functional unit formed by the interaction and interdependence of biological and non-biological components through material circulation and energy flow in a certain time and space. The ecosystem has certain structural characteristics. A basic understanding of the family ecosystem theory derived from the ecosystem is that there is a mode of interaction in the family system. The interaction of family members occurs at the same time. The behavior of one member will affect the behavior, cognition, and emotion of other members, and will also lead to their reflection on the behavior, cognition, and emotion. Some changes made by one member will lead to different changes in other members [9]. According to the degree of interaction between individuals and the environment, the family ecosystem is divided into four environmental systems from the inside out, namely, the micro-system, which includes individuals and has the most direct interaction with individuals, such as family. Mesosystem refers to the relationship between family and work unit. Exosystem is the process and connection between multiple environments. At least one of these environments does not include individuals, but the events occurring in them will have an impact on the process of interaction between micro systems. Macrosystem refers to the possible consistency in content and form of various lower level ecosystems at the sub-cultural level. The above four interacting environmental systems are like Russian dolls, each of which is nested in an adjacent level, and jointly affect the development of family members.

Under the premise of family interaction, the negative emotions and marital feelings between husband and wife overflow, and then predict the negative interaction with children, which is called the Spillover [10, 11]. There is a strong relationship between marital disharmony and children’s adaptive function, from physical health to emotional expression and cognitive dysfunction [12]. In one study, depression scores of couples were used as a moderator to measure the spillover effect of marital quality. Both father and mother’s depression weakened the link between the quality of relationship between their spouses and children [13]. For a long time, parents have been playing the role of childcare in the family system, and will also produce stress and burnout in the process of working to achieve parenting, of which emotional exhaustion is considered to be an important part. According to a research report, parental burnout in the process of childcare directly or indirectly aggravates spouse’s job burnout [14]. So under the pressure of childcare, will raising more children further aggravate the pressure of parents’ marriage? A notable study [15] investigated the well-being of married couples in Poland from the perspective of the number of children they had (notably, Poland has a very low total fertility rate of 1.4). The study showed that the first child was a significant positive predictor of the mother’s subjective well-being; that is, the first child significantly increased the mother’s subjective well-being. However, fathers experienced a smaller increase in happiness with the birth of their first child. Meanwhile, the second child did not produce additional well-being for the couples. At the same time, Twenge et al. [16] found that having a higher number of children reduces marital satisfaction in married couples. However, the generalizability of these findings is limited, as the vast majority of studies have been conducted in Western countries with an individualistic culture. Nonetheless, a meta-analysis conducted by Dillon and Beechler in 2010 included 15 studies from collectivist cultures, and showed a negative relationship between the number of children and marital satisfaction [17]. Meanwhile, Onyishi et al. [18] conducted a study in Nigeria, a developing country that also has a collectivist culture. The results indicated a positive relationship between the number of children and marital satisfaction between parents; the number of children was the strongest predictor of marital satisfaction even when compared to other variables, such as wealth and education. Li Qiang et al. [19] found that the higher the number of children, the lower the life satisfaction of middle-aged couples. Moreover, for individuals with severe depression, as the number of children increased, parents’ life satisfaction did not change significantly but their depression levels increased significantly. Furthermore, number of children was shown to have a lower negative prediction of self-rated life satisfaction in older parents than in middle-aged parents. Thus, in both Western and Eastern societies and in low- and high-fertility countries, having more children seems to have negative effects on parents. China’s 2021 census data showed that its total fertility rate was 1.3, an extremely low level. Due to delays in marriage, the fertility rate will continue to be depressed for decades to come. While the three-child policy, implemented in 2021, now allows a couple to have three children, future increases in the married birth rate remain uncertain [20]. In a low-fertility society, the willingness and ability to have more children can be seen as a measure of social welfare and marital happiness.

Based on the family system theory, there is an interactive relationship between husband and wife in the family, especially the extreme cognitive style and emotional changes of the husband and wife will affect the partner’s behavior [9]. The personality characteristics of the husband and wife will affect the partner’s marital quality and its spillover effect, which will hinder the establishment of a healthy family stability [21]. “Neuroticism” is characterized by excessive worry, easily becoming nervous, and a poor ability to cope with stress. It may be a significant personality factor related to marital relationships and mental health. Scholars found that neuroticism was significantly and positively correlated with depression, negatively correlated with marital happiness, and negatively correlated with trust in strangers on the Big Five Personality Inventory. A review of 18 correlational studies in Iran found that couples with higher levels of neuroticism were less satisfied with their marriages [22]. In a longitudinal study by Fisher and McNulty with 72 couples in Ohio, USA, high levels of neuroticism predicted lower marital satisfaction one year later [23]. Personality reveals itself in the way couples cope with life and behavioral characteristics, and it further affects their health. Paranoia and neuroticism can cause tension in marital relationships and easily trigger marital conflicts. The mental health of both spouses is highly correlated with the other’s poor self-regulation ability (i.e., being prone to anxiety and paranoia), which can make the spouse’s family life more stressful, resulting in depression and other adverse emotions [24].

Family size is often recognized as a risk factor for parents’ well‐being, given the association between family size and higher levels of parenting stress [25]. The number of family members brings about changes in family structure, so the family size changes the quality of marriage and family stability by causing the spillover effect of marriage satisfaction [7]. In an explanatory model generated by Farkas C et al. [26] for self-efficacy and maternal stress, the results showed that family size was relevant in explaining maternal self-efficacy and anxiety. A recent study by Fiorillo et al. [27] shows that living with more family members was a protective factor against the development of mental symptoms during the lockdown imposed due to the COVID-19 pandemic, which indicates that living with more family members were beneficial to the physical and psychological health of individuals.

Data about the effects of marital relationships on mental health are often paired. Most of the studied variables of a couple have an interactive relationship. This non-independent relationship is due to the fact that a person’s characteristics or behavior will affect his or her partner. Analyzing this relationship at the level of the individual will increase the probability of type I and type II errors in statistical hypothesis testing and fail to account for the variable of nonindependence in interpersonal relationships. The Actor–Partner Interdependence Model (APIM), proposed by Kenny et al. [28] is used by scholars around the world to explore actor and partner effects in a marital relationship by studying the effect of the individual’s independent variables on the dependent variable (e.g., regarding the actor effect, the effect of the husband’s satisfaction on his depression) and the effect of spouse independent variables on the dependent variable (e.g., regarding the partner effect, the effect of the wife’s satisfaction on the husband’s depression). We adopt the convention that the partner effect refers to the explained variable. In other words, the effect of husband’s marital satisfaction on wife’s depression is called wife’s partner effect. The effect of wife’s marital satisfaction on husband’s depression is called husband’s partner effect. On the basis of APIM, the Actor–Partner Interdependence Moderation Model (APIMoM) adds another moderating variable, *M*. This enables the study of any interaction between *M* and the predictor variable that could affect the direction and intensity of the effect of the predictor variable on the outcome variable [29]. The APIM can be used if the effect of affinity on marital satisfaction is studied. Meanwhile, the APIMoM can be used if researchers want to understand whether the effect may vary according to how long the couples have been married. In this model, data pairs are categorized as distinguishable or indistinguishable, depending on the meaningful variable that distinguishes two members within the pair. In terms of gender, two members of a couple are distinguishable, but two members of a same-sex couple are indistinguishable. From the perspective of family ecosystem theory [9], family unit is a perfect whole, in which husband and wife are both elements of the system. In the family system, husband and wife jointly bear the responsibility and obligation of raising children and maintaining the quality of marriage. They maintain the integrity of the family through their interaction with each other. On the one hand, the number of children and family members represent the characteristics of the family, of the system in which husband and wife live together. On the other hand, husbands and wives, as elements of the family system, may also have individual characteristics that affect each other’s mental health. Personality is a relatively stable psychological characteristic or behavior mode of individual, and neuroticism is the most negative influence of many personality traits on the quality of marital relationship between husband and wife [30]. It mainly affects the quality of marital relationship through interaction processes, such as negative interpersonal behavior, emotional expression, etc. Personality as an internal factor, under the same conditions, different personality characteristics will lead to different morbidity [31]. Neurotic personality is related to the age of onset, duration and lifetime prevalence of female depression, and also has an impact on the quality of marriage [32]. Therefore, the two moderating variables of number of family members and number of children can be grouped under the category of family structure, and the other one moderating variable of neuroticism can be seen as the category of individual characteristics.

In the process of data analysis, according to whether the value between husband and wife is different, they can be divided into three categories, namely, intra-pair variables, inter-pair variables and mixed moderator variables. The intra-pair moderator variables are the variables that distinguish between the paired data, such as gender. Inter-pair moderator variables have the same value for the given pair and vary between different pairs, such as the number of children, marital age of couples, and family economic level. Mixed moderator variables are different within and between pairs, that is, individual independent variables, such as the degree of trust between husband and wife and neurotic personality traits. Among all the moderator variables, gender is used to distinguish the paired data between husband and wife and is included in the two-intercept model. It is involved in each moderating model to distinguish the actor effect and the partner effect.

One frequently studied effect of APIM in marital relationships is the gender effect. This is because women play the role of caregivers more than men do and provide more support in the home environment [33], which means that they experience more life burdens. In addition, women’s need for companionship is more intense than that of men [34]. This is supported by Ayotee [35], who found that the wife’s depressed mood was affected by the husband’s mood and, conversely, that the depressed mood of husbands was not significantly affected by that of the wives. In contrast, Ruthing [36] reported that the husband’s well-being was significantly predicted by the wife’s health status but not by the husband’s health status. In addition, Bourassa et al. [37] did not find a gender effect in the significant association between spouse health and personal quality of life.

In this study, the APIMoM was used to analyze the effect of marital satisfaction on couples’ self-rated depression. The number of children and the number of family members were used as interpair moderator variables, and neuroticism was used as a mixed regulatory variable to analyze whether moderator variables affected the direction and intensity of marital satisfaction on self-rated depression. In addition, two-intercept model analyses were conducted to examine the gender effect.

Based on tenets of family ecosystem theory, past research, and APIMoM, our general hypotheses contend that:

### Hypothesis 1 (H1)

Marital satisfaction (i.e., the actor effect) and spousal marital satisfaction (i.e., the partner effect) are negatively associated with depression levels.

### Hypothesis 2 (H2)

A weaker negative association between one’s marital satisfaction and spousal depression levels (i.e., the partner effect) are found among people with more children than among those with fewer children.

### Hypothesis 3 (H3)

A stronger negative association between one’s marital satisfaction and depression levels (i.e., the actor effect) and a stronger negative association between spousal marital satisfaction and one’s depression levels (i.e., the partner effect) are found among people with more family members than those with fewer family members.

### Hypothesis 4 (H4)

A weaker negative association between one’s marital satisfaction and depression levels (i.e., the actor effect) and a weaker negative association between spousal marital satisfaction and one’s depression levels (i.e., the partner effect) are found among people with higher neuroticism than those with lower neuroticism.

## Materials and methods

### Participants

The data used in this study were derived from Peking University’s China Family Panel Studies (CFPS) questionnaire dataset. The CFPS is a national and comprehensive social tracking survey project. It collects and tracks data over the long term at individual, family, and community levels. The survey content includes village/household profiles, family relationships, family economy, housing and facilities, education, marriage, health, attitudes, cognitive abilities, and social interaction [36]. This study used CFPS data from 2018; the coding of family and marital relations in this data enables the matching of information related to each couple. During the data sorting process, observations that involved missing values, wrong answers, and refusal to answer were eliminated, and data on 9,560 couples were ultimately collected. It should be noted that the variables of each couple are matched; for example, the observation of the wife contains the variable representing the depression of the husband.

### Measures

#### Center for epidemiologic studies depression scale

The depression of the respondents was measured by asking them to indicate the frequency with which they had experienced the following situations in the past week: “I feel depressed,” “I feel strained doing anything,” “My sleep is not good,” “I feel happy,” “I feel lonely,” “I live a happy life,” “I feel sad,” and “I feel that life cannot continue.” Respondents were asked to respond by scoring the items using a scale ranging from 1 to 4 (1 = hardly ever, less than one day; 2 = sometimes, 1–2 days; 3 = often, 3–4 days; 4 = most times, 5–7 days). Two of the statements are scored in reverse (“I feel happy” and “I live a happy life”). The higher the score was, the more severe was their depression. The Cronbach’s alpha coefficient was 0.75.

#### Marital satisfaction scale

To measure how satisfied couples were with their marital lives and spouses, three questions were considered: “In general, how satisfied are you with your current marital life?,” “How satisfied are you with your spouse’s financial contribution to the family?,” and “How satisfied are you with your spouse’s contribution to the family in terms of household chores?” Participants indicated their marital satisfaction on a 5-point scale from 1 (not satisfied at all) to 5 (completely satisfied). The more comfortable the marriage, the higher the score. The Cronbach’s alpha coefficient was 0.749.

From 19,120 valid data, 50% of the sample data were randomly selected for exploratory factor analysis, and the other half for confirmatory factor analysis. For exploratory factor analysis, the KMO test and Bartlett’s sphericity test results were used to determine whether the scale was suitable for principal component analysis. According to relevant standards, KMO value > 0.6 is suitable for factor analysis, and 0.9 and above is very suitable for factor analysis. Bartlett’s sphericity test was significant, indicating the possibility of sharing factors among items. The KMO value of the marriage satisfaction scale used was 0.693, indicating that there was no significant difference between the items. At the same time, Bartlett’s sphericity test results were significant (χ^2^ = 7249.660, *p* < 0.001), indicating that it is very suitable for factor analysis. The principal component analysis and the varimax orthogonal rotation were used to obtain the final factor load matrix. One factor was extracted mainly reflecting the satisfaction of individuals with their marital life and spouse’s family contributions, and was named “marital satisfaction”, which included three items. The remaining half of the data was used for confirmatory factor analysis. The theoretical model of the marriage satisfaction scale, including three first-order factors, were the three items corresponding to the scale. The fitting indicators were RMSEA = 0.000, RMR = 0.000, GFI = 1.000, NFI = 1.000, CFI = 1.000. The results of confirmatory factor analysis showed that the fitting indicators of the model met the acceptable standards, and finally used the sum of the scores of the three items to construct the variable marital satisfaction.

#### Brief Version 5 personality scale

We used two items from the neuroticism dimension of the Brief Version 5 Personality Scale: “often worried” and “easily become nervous.” Participants rated their neuroticism on a 5-point scale from 1 (completely disagree) to 5 (completely agree). The higher the score, the higher their level of emotional instability.

#### Control variables

Since the retirement status and the household’s economic situation contribute to the spouses’ mental health [38], we checked the database and selected the following demographic variables as the control variables. Age, region, education level, ethnic composition (i.e., Han and ethnic minorities), employ (i.e., unemployment, employment, and drop out of the labour market), health, smoking frequency (whether smoke in the past month), monthly after-tax income, and medical expenses.

### Statistical analysis

#### Descriptive analysis and data preprocessing

Comparisons of demographic characteristics, depression, marital satisfaction, and neuroticism for men and women were performed via the McNemar test and an independent samples t test. Pearson’s correlation coefficient was calculated to investigate the bivariate relationship among the study variables. If the raw data is directly used for analysis, it will highlight the role of indicators with higher values in the comprehensive analysis, and relatively weaken the role of indicators with lower values. Standardization can avoid the analysis error of some extreme values. Therefore, the data is pretreated by z-core standardization (eg., marital satisfaction, depression, neuroticism) before establishing the regression model. Because the standard Z-score allows variables to obtain the same measurement scale, the mean is 0 and the standard deviation is 1, which better solves the comparability problem among data indicators and is more suitable for comprehensive comparative analysis.

#### APIMoM

We used STATA to collate and merge the database, and then used SPSS software for descriptive statistical analysis and APIMoM model analysis. The APIMoM can be implemented by a multilevel model (MLM), which uses maximum likelihood estimation for analysis. Introduce the product term of the predictive variable and the moderator that may have interaction in the equation, and then test whether the product term is statistically significant, also known as the linear-by-linear interaction term. The interaction approach model and the two intercept model [29] are used to solve the parameters. The former can directly show whether the actor–partner effect will be regulated by *M* (the moderator variable), and the latter can be used to understand the regulation of moderator variables on the actor–partner effect of the two members.

**Fig. 1:**
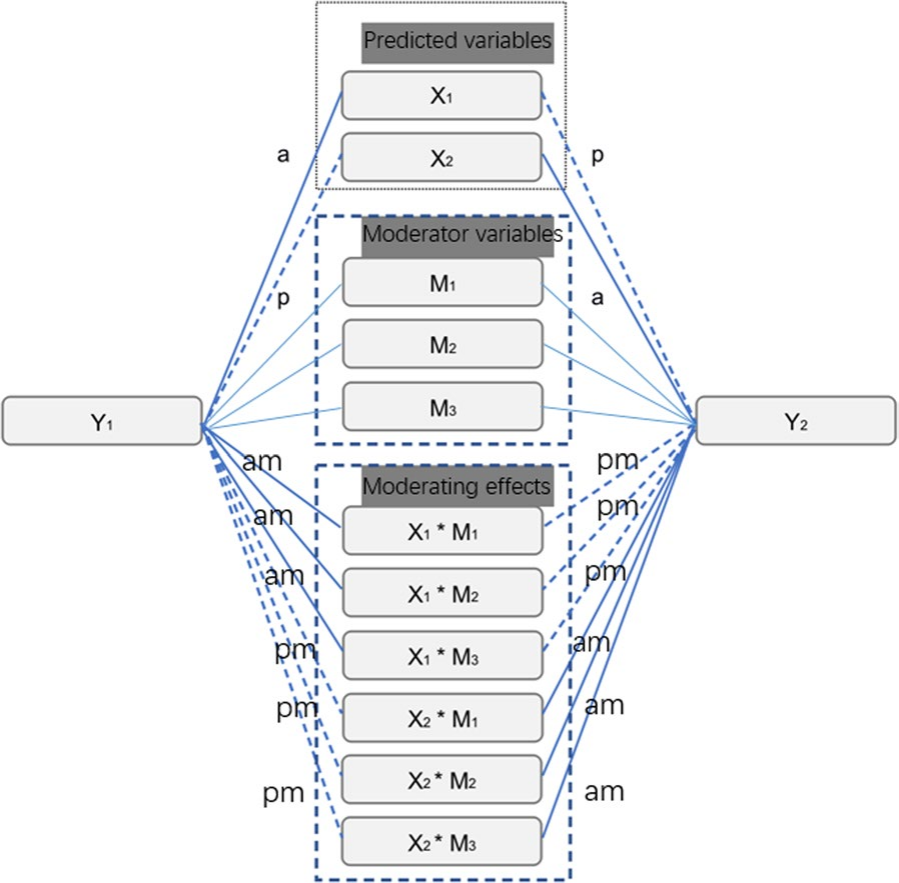
APIMoM model structure diagram. *Notes* X1: Husband’s marital satisfaction, X2: Wife’s marital satisfaction, Y1: Husband’s depression, Y2: Wife’s depression, M1: Number of family members, M2: Number of family members* number of children, M3: Couple’s neuroticism. A and p represent the actor effect and partner effect of individuals; am and pm represent the effect of the interaction between the individual’s predicted variable and the moderator variable on their own outcome variables and spouse outcome variables.

The assumption adopted by APIMoM is that the moderating effect is linear, that is, as the moderator variable changes, the change degree of the influence of the predicted variable on the outcome variable is quantitative, and the moderating effect can be predicted by the interaction term. When the interaction term is included in the model, the main effect of the variable is also included in the model [29]. The structure diagram of APIMoM is shown in Fig. 1, and its information supplement is included at the bottom of the figure.

We first used the main effects model, which included control variables, intra-pair moderator variables (gender) and predicted variables, to analyze whether the actor and partner effects of husbands and wives were significant. Then, the inter-pair moderator variables (number of children and number of family members) were added to observe their moderating effects on the couple actor effect and the partner effect. Finally, a mixed moderator variable (neuroticism) was added to the MLM. The distinguishability of the mixed moderator variable was the most complicated for the APIMoM. The modes of moderation were as follows:

1. the women’s neuroticism moderates women’s actor effects,
2. the men’s neuroticism moderates women’s actor effects,
3. the women’s neuroticism moderates women’s partner effects,
4. the men’s neuroticism moderates women’s partner effects,
5. the women’s neuroticism moderates men’s actor effects,
6. the men’s neuroticism moderates men’s actor effects,
7. the women’s neuroticism moderates men’s partner effects,
8. the men’s neuroticism moderates men’s partner effects.

We observed whether the results of three important interaction terms (neuroticism, actor–partner effect, and gender) were significant to determine whether neuroticism affected the direction and strength of the effect of marital satisfaction on self-rated depression.

#### Statistical software

All preliminary analyses were carried out using Stata v. 16.0 (Stata Corporation LLC, College Station, USA), and the APIMoM analysis was carried out using IBM SPSS Statistics for Windows, Version 23.0 (IBM Crop. Armonk, NY, USA).

## Results

Table 1 shows the demographic variables of the subjects, including the study variables. The results show that the average age of husbands is 1.89 years older than their wives. The average age of the wives was 49, with ages ranging from 20 to 86. The average age of the husbands was 51, with ages ranging from 22 to 93. The wives’ self-rated depression score was 1.03 points higher than that of their husbands on average, indicating that the wives may experience more severe depression. The scores of marital satisfaction and neurotic personality of husbands and wives were similar. In addition, there are significant differences in other demographic characteristics of couples, and these variables are included in the regression equation as the control variables for analysis.

**Table 1:** Demographic characteristics of the male and female dyads (n = 9560 couples)

	Husband	Wife	Statistics	P
Age(years)	50.87 ± 13.93	48.98 ± 13.69	t = − 9.477	< 0.001***
Marital satisfaction	13.75 ± 1.95	12.61 ± 2.55	t = 0.049	0.961
Neuroticism	6.21 ± 1.97	6.89 ± 1.90	t = 0.063	0.949
Depression	12.86 ± 3.70	13.89 ± 3.97	t = 18.577	< 0.001***
Health	3.01 ± 1.18	3.20 ± 1.24	t = 10.953	< 0.001***
Region			χ^2^ = 22.792	< 0.001***
Rural	6947 (72.2)	7233 (75.2)		
Nonrural	2678 (27.8)	2392 (24.8)		
Educational level			χ^2^ = 652.587	< 0.001***
Illiteracy	1419 (14.7)	2881 (29.9)		
Primary school	3981 (41.4)	3110 (32.3)		
Middle school	2210 (23.0)	1835 (19.1)		
High school/junior college	1285 (13.4)	1128 (11.7)		
Undergraduate college	322 (3.4)	303 (3.2)		
Missing	407 (4.2)	368 (3.8)		
Ethnicity			χ^2^ = 7.823	< 0.05*
Han	9054 (94.1)	8994 (93.4)		
Ethnic minorities	571 (5.9)	631 (6.6)		
Partner’s Employ			χ^2^ = 500.634	< 0.001***
Employment	8085 (84.0)	6797 (70.6)		
Unemployment	68 (0.7)	74 (0.8)		
Drop out of the labour market	1472 (15.3)	2754 (28.6)		
Partner’s Smoking frequency (whether smoke in the past month)			χ^2^ = 6939.725	< 0.001***
No	4063 (42.2)	9371 (97.4)		
Yes	5562 (57.8)	254 (2.6)		
Partner’s Medical expenses			χ^2^ = 164.549	< 0.001***
X ≤ 1000	6841 (71.1)	6096 (63.3)		
1000 < X ≤ 5000	1591 (16.5)	2153 (22.4)		
5000 < X ≤ 30,000	956 (9.9)	1199 (12.5)		
X > 30,000	237 (2.5)	175 (1.8)		
Partner’s Monthly after-tax income			χ^2^ = 897.083	< 0.001***
X ≤ 3000	7206 (74.9)	8765 (91.1)		
3000 < X ≤ 10,000	2322 (24.1)	826 (8.6)		
X > 10,000	97 (1.0)	34 (0.3)		
Family size	4.20 (1.66)	–		
Number of children	1.78 (0.78)	–		

Values are given as “Mean ± SD” or “n (%)”, *p < 0.05, ***p < 0.001

Table 2 presents the correlation coefficients between marital satisfaction, depression, neuroticism, number of children, and number of family members between the members of the couple dyad. Pearson’s correlation was used to examine the correlation of variables between husbands and wives. The results showed that the husbands’ marital satisfaction was negatively correlated with their own depression scores and that of their wives. Similarly, the wives’ marital satisfaction was negatively correlated with their own depression scores and that of their husbands.

**Table 2:** Correlation coefficients among variables in the husband and wife dyads (n = 9560 couples)

	1	2	3	4	5	6	7	8
*Husband*								
1. Marital satisfaction	1.000							
2. Neuroticism	− 0.011	1.000						
3. Depression	− 0.211***	0.281***	1.000					
*Wife*								
4. Marital satisfaction	0.233***	− 0.006	− 0.139***	1.000				
5. Neuroticism	− 0.022**	0.122***	0.076***	− 0.034***	1.000			
6. Depression	− 0.109***	0.100***	0.290***	− 0.220***	0.254***	1.000		
7. Number of children	− 0.032***	0.030***	0.066***	0.018*	0.068***	0.087***	1.000	
8.Number of family members	− 0.051***	0.055***	0.015	− 0.033***	0.055***	0.002	0.192***	1.000

*p < 0.05, **p < 0.01, ***p < 0.001

Table 3 shows the analysis results of control variables, main effect models and actor partner effects. As for the actor effect, husbands and wives with high marital satisfaction have lower depression scores and experience less depression (b =  − 0.19, *P* < 0.001; b =  − 0.21, *P* < 0.001, respectively). In other words, after taking into account the effect of spouse, for each increase in marital satisfaction of husband and wife by 1 point, their self-rated depression scores decreased by 0.19 and 0.21 points respectively. As for the partner effect, men with high marital satisfaction of their wives have lower depression scores (b =  − 0.09, *P* < 0.001), while women with high marital satisfaction of their husbands have lower depression scores (b =  − 0.06, *P* < 0.001).

**Table 3:** The results of the control variables and main effects model analysis (n = 9560 couples)

APIM parameters	Role	Estimate	SE	95%CI
Intercept		− 0.14*	0.06	− 0.25, − 0.03
Age		− 0.006***	0.0007	− 0.007, − 0.004
Region		− 0.09***	0.009	− 0.10, − 0.07
Educational level		− 0.06***	0.008	− 0.08, − 0.05
Ethnic composition		0.02	0.03	− 0.04, 0.07
Employ		− 0.04***	0.009	− 0.06, − 0.02
Health		0.22***	0.006	0.20, 0.23
Smoking frequency		0.07***	0.02	0.04, 0.11
Monthly after-tax income		− 0.03	0.02	− 0.07, 0001
Medical expenses		0.10***	0.01	0.08, 0.12
Gender		0.02*	0.008	0.0007, 0.03
	Husband			
Marital satisfaction (actor effect)		− 0.19***	0.01	− 0.21, − 0.17
Wife’s marital satisfaction (partner effect)		− 0.09***	0.01	− 0.11, − 0.07
	Wife			
Marital satisfaction (actor effect)		− 0.21***	0.01	− 0.23, − 0.19
Husband’s marital satisfaction (partner effect)		− 0.06***	0.01	− 0.08, − 0.04

*p < 0.05, ***p < 0.001

Table 4 shows the results of APIMoM analysis, in which the number of family members is an moderator variable. The results showed that the number of family members had a positive moderating effect on the results of the wife’s partner effect (b = 0.02, *P* < 0.001). With the increase of the number of family members living together, the negative correlation between husband’s marital satisfaction and wife’s depression degree would be amplified. In other words, on the premise that the score of marital satisfaction of the husband increases by the same score, the depression score of the wife who lives in a larger family size will further decrease.

**Table 4:** The results of the APIM with the number of family members as a moderating variable (n = 9560 couples)

APIM parameters	Role	Estimate	SE	95%CI
Intercept		− 0.11	0.06	− 0.23, 0.01
Number of family members		− 0.006	0.005	− 0.01, 0.003
Actor’s marital satisfaction * Number of family members		0.005	0.004	− 0.002, 0.01
Partner’s marital satisfaction * Number of family members		0.01*	0.004	0.002, 0.02
	Husband			
Marital satisfaction (actor effect)		− 0.23***	0.03	− 0.28, − 0.17
Wife’s marital satisfaction (partner effect)		− 0.10	0.03	− 0.16, − 0.05
Number of family members * Marital satisfaction (actor effect)		0.009	0.006	− 0.003, 0.02
Number of family members * Wife’s marital satisfaction (partner effect)		0.002	0.006	− 0.01, 0.01
	Wife			
Marital satisfaction (actor effect)		− 0.20***	0.03	− 0.26, − 0.15
Husband’s marital satisfaction (partner effect)		− 0.16***	0.03	− 0.22, − 0.10
Number of family members * Marital satisfaction (actor effect)		− 0.0006	0.006	− 0.01, 0.01
Number of family members * Husband’s marital satisfaction (partner effect)		0.02***	0.006	0.01, 0.03

*p < 0.05, ***p < 0.001

Table 5 shows the results of APIMoM analysis, including the number of family members and the number of children as moderators. The results showed that couples who have more children have higher depression scores (b = 0.06, *P* < 0.05). The number of children has a negative moderating effect on the results of partner effect of husbands and wives (b =  − 0.06, *P* < 0.01; b =  − 0.06, *P* < 0.01, respectively), on the premise that their marital satisfaction increased by the same score, the effect of spousal depression score reduction of men and women who raised more children was more implicit. The last is about the moderating effect of the interaction between the number of family members and the number of children. The interaction has a positive moderating effect on the wife’s partner effect (b = 0.01, *P* < 0.001). With the increase of the interaction item value, the negative correlation between husband’s marital satisfaction and wife’s depression will be amplified, which means that the increase of husband’s marital satisfaction score will more effectively reduce the wife’s depression score.

**Table 5:** The results of the APIM with the number of family members and the number of children as moderators (n = 9560 couples)

APIM parameters	Role	Estimate	SE	95%CI
Intercept		− 0.16*	0.08	− 0.31, − 0.01
Number of family members		− 0.01	0.01	− 0.03, 0.01
Number of children		0.06*	0.02	0.01, 0.11
	Husband			
Number of children * Marital satisfaction (actor effect)		− 0.005	0.02	− 0.04, 0.03
Number of children * Wife’s marital satisfaction (partner effect)		− 0.06**	0.02	− 0.1, − 0.02
Number of children * Number of family members * Marital satisfaction (actor effect)		0.004	0.003	− 0.002, 0.009
Number of children * Number of family members * Wife’s marital satisfaction (partner effect)		0.003	0.003	− 0.003, 0.009
	Wife			
Number of children * Marital satisfaction (actor effect)		0.02	0.02	− 0.02, 0.06
Number of children * Husband’s marital satisfaction (partner effect)		− 0.06**	0.02	− 0.10, − 0.02
Number of children * Number of family members * Marital satisfaction (actor effect)		− 0.002	0.003	− 0.008, 0.004
Number of children * Number of family members * Husband’s marital satisfaction (partner effect)		0.01***	0.003	0.005, 0.02

*p < 0.05, **p < 0.01, ***p < 0.001

Table 6 shows the results of APIMoM analysis with neuroticism as a mixed moderator variable. The results showed that couples with high neuroticism scores predicted high depression scores of themselves and their spouses. In the subsequent two-intercept model, the wife’s neurotic personality score has a negative moderating effect on the wife’s actor effect (b = − 0.03, *P* < 0.001). With the increase of the wife’s neurotic personality score, the negative correlation between the wife’s marital satisfaction score and depression score will be weakened.

**Table 6:** The results of APIM Analysis with Neuroticism as a moderating variable (n = 9560 couples)

APIM parameters	Role	Estimate	SE	95%CI
Intercept		− 0.26***	0.05	− 0.37, − 0.16
Actor’s neuroticism * gender		0.02***	0.007	0.01, 0.04
Partner’s neuroticism * gender		− 0.02*	0.007	− 0.002, 0.02
	Husband			
Neuroticism		0.28***	0.01	0.26, 0.29
Wife’s neuroticism		0.04***	0.01	0.02, 0.05
Marital satisfaction (actor effect)		− 0.19***	0.01	− 0.20, − 0.17
Wife’s marital satisfaction (partner effect)		− 0.09***	0.01	− 0.11, − 0.07
Neuroticism * Marital satisfaction (actor effect)		− 0.007	0.01	− 0.03, 0.01
Wife’s neuroticism * Marital satisfaction (actor effect)		− 0.004	0.01	− 0.02, 0.02
Neuroticism * Wife’s marital satisfaction (partner effect)		− 0.02	0.01	− 0.04, 0.0007
Wife’s neuroticism * Wife’s marital satisfaction (partner effect)		0.02	0.02	− 0.005, 0.03
	Wife			
Neuroticism		0.24***	0.01	0.22, 0.26
Husband’s neuroticism		0.07***	0.01	0.05, 0.09
Marital satisfaction (actor effect)		− 0.20***	0.01	− 0.22, − 0.18
Wife’s marital satisfaction (partner effect)		− 0.06***	0.01	− 0.08, − 0.04
Neuroticism * Marital satisfaction (actor effect)		− 0.03***	0.01	− 0.05, − 0.01
Husband’s neuroticism * Marital satisfaction (actor effect)		− 0.003	0.01	− 0.02,0.02
Neuroticism * Husband’s marital satisfaction (partner effect)		0.01	0.01	− 0.009,0.03
Husband’s neuroticism * Husband’s marital satisfaction (partner effect)		0.004	0.01	− 0.02,0.02

*p < 0.05, ***p < 0.001

Marital satisfaction is a relatively subjective measurement index, and the evaluation criteria of the observation objects are different. For the purpose of robustness analysis, the explanatory variable “marital satisfaction” was recoded, with 1–3 points as 0 and 4–5 points as 1; 1–2 points are scored as 0, and 3–5 points are scored as 1. 36.75% of women and 59.0% of men answered “completely satisfied” to the all three questions. The t-test results of the predictive variables before recoding were not significant, while the chi-square test results after recoding showed that the marital satisfaction scores of couples were significantly different (χ^2^ = 1073.497, *P* < 0.001; χ^2^ = 631.623, *P* < 0.001, respectively). The recoded variables were included in the regression equation analysis, and the control variables, main effects and moderating effects of the model (the number of family members and children as the moderator variables) were compared. Their influence coefficients, influence directions and significance were similar. Among them, there was still a negative correlation between marital satisfaction and depression, and the significance of the actor effect and partner effect of husband and wife in the above model was also consistent with the previous article. Under the influence of the interaction between husband and wife, the significant results of the interaction terms of these moderator variables have not changed, indicating that the results of this study have certain robustness. In the model of neurotic personality as a moderator, there were additional significant items. The influence coefficient and significance of the interaction items did not change after the second way of re-coding for analysis. In the first way of re-coding for analysis, the wife’s neurotic personality had a positive moderating effect on the husband’s partner effect (b = 0.04, *P* < 0.05), except that the significant items of the original model were still maintained. That is to say, with the increase of the wife’s neurotic personality score, the negative correlation between the wife’s marital satisfaction score and the husband’s depression will strengthen, and the negative correlation between the wife’s marital satisfaction score and the husband’s depression will weaken. This suggests that if the wife is often in a tense mood in her marriage life and shows concern about the quality of marriage, this phenomenon seems to have the opposite effect on the depression of the wife and husband. The items about neuroticism score in this paper come from the simplified version of the Brief Version 5 Personality Scale, and the reverse question item was removed, and there are two items in total. Therefore, it is necessary to use a more complete scale to measure this variable in future research, and explore the moderating effect in the quality of marriage again to find the phenomenon behind the unstable results.

## Discussion

The APIMoM provides moderator variables that can be used to investigate whether the strength of actor-partner effects in pair members changes with the addition of specific exposure factors. The quantity and direction of the effect between explanatory variables and outcome variables in the study may be altered or even reversed by newly added distinguishing characteristics (i.e., the moderator variable), which may help to further examine the validity of the effect [39]. Finally, it is possible that the inclusion of selections and studies is not the true moderator variable; instead, other variables related to it, such as gender, may have a partial moderating effect. More meaningful moderator variables such as height, income, or social status may be hidden behind these features [40]. The APIMoM has been used in several studies, most of which focus on mental health issues in marriage and love relationships [7], as well as studies on doctor–patient interactions [41].

The descriptive statistics showed that the wife’s self-rated depression score was 1.03 points higher than that of the husband, and the score of marital satisfaction and neurotic personality were similar between the husband and wife. Consistent with the results of two studies conducted in Poland [42] and Pakistan [43], wives are more depressed and dissatisfied with marriage than husbands. The same research results were reported in a study by Peterson et al. [44]. There was no significant difference in marital satisfaction between wife and husband.In addition, general population studies have shown that marital dissatisfaction and anxiety are more common among women than among men [45]. In terms of measures to prevent depression, women’s mental health should be given more priority than that of men.

The results in Table 3 confirm Hypothesis 1. Members’ marital satisfaction was significantly negatively correlated with their own and spouse’s depression levels. However, several previous studies [7, 46] reported an partially significant opposite pattern in the partner effect. The results of the current study showed that maintaining a good marital relationship, making significant economic and household contributions, and being relatively satisfied in marriage effectively improved and prevented depression among couples. Based on the family system theory [9], there is an interactive relationship between husband and wife. Their dissatisfaction with marriage and their passive handling of housework and raising children will affect their spouses’ cognitive and emotional changes. Due to the high emotional correlation between husbands and wives, addressing wives’ dissatisfaction and suspicions, and maintaining their marital satisfaction can effectively reduce and prevent the occurrence of depression in their husbands.

The results listed in Table 4 are consistent with Hypothesis 3, living with more family members magnified the negative correlation between husbands’ marital satisfaction and wives’ depression. Living with more family members reduced financial burdens and housework pressures for couples, improved marital satisfaction, strengthened couples’ positive emotions, and ultimately effectively reduced anxiety and depression among wives. On the premise that the score of marital satisfaction of the husband increases by the same score, the depression score of the wife who lives in a larger family size will further decrease.

Consistent with previous scholars’ conclusions, the greater the number of children, the more severe the depression may be among members of a couple [18]. The results listed in Table 5 are consistent with Hypothesis 2. On the premise that their marital satisfaction increased by the same score, the effect of depression score reduction of men and women who raised more children was more implicit. Under the background of family ecosystem theory, when the depression and dissatisfaction of both parties in the marriage are transferred to raising more children, overflow will occur. It is speculated that this situation is due to marital dissatisfaction and depression, and the inability to carry out sensitive and responsive childcare. According to the compensatory hypothesis put forward by some scholars, parents compensate their dissatisfied marriage by investing more time and energy in high-quality parenting [47]. Although the compensatory hypothesis has received less attention in theory, the motivation to invest more time in parent–child relationship has been interpreted as a means to realize the unsatisfied need for love and support within the family. This can be proved by the negative correlation between the quality of marriage and the quality of parent–child relationship. What cannot be ignored is that the challenge of raising children and stepchildren is more prominent in the stepfamilies, which usually faces more and more unacceptable difficulties in raising children [48]. The pressure experienced by parents in stepfamilies can also cause spillover effects, leading to negative emotions such as disappointment of their partners [14].

In contrast, we found that living with more children and more family members (e.g., their children’s grandparents, aunts) not only alleviates depression in wives, but also predicts lower depression in couples. Living in a larger family with more children amplified the negative relationship between husbands’ marital satisfaction and wives’ depression levels. The increase of husband’s marital satisfaction score will more effectively reduce the wife’s depression score. In a low-fertility society, the willingness and ability to have more children can be seen as a measure of social welfare and marital happiness. In the context of China’s implement of the three-child policy, millions of families have welcomed more newborns. Thus, we suggest that having multiple children and living with relatives may be good for the mental health of both husbands and wives. This is also consistent with the conclusion of Fiorillo et al. [27].

In married life, excessive worry and tension between husbands and wives will undoubtedly increase suspicion, quarrels, and contradictions [24]. Regardless of which spouse had a higher neuroticism score, both spouses were more depressed. Consistent with Hypothesis 4, neuroticism among wives minimized the negative correlation between their own marital satisfaction and depression. On the premise that marriage satisfaction increased by the same score, the effect of depression score reduction of women with high neuroticism score was more recessive. Wives who are overly worried and nervous tend to be more suspicious and quarrelsome in their marriages, their nervousness predicts a high depression level. This suggests that in the process of preventing couples’ depression, mental health workers should not only emphasize the promotion of marital satisfaction but also consider the neurotic personality of the couple, especially the wives, and conduct special treatments and preventive measures to more effectively promote the mental health of couples.

Robustness analysis shows that the results of this study have certain robustness. The new significant results show that the further classification of the scores of the subjects in the process of data collation has a certain impact on the analysis results. When measuring people’s marital satisfaction in subsequent similar studies, the score can be divided into more detailed scores, such as 10 points, or even items, such as 6 points and 8 points. The author suggests adding a description of subjective feelings, for example, 3 points of satisfaction can be added “husband or wife often neglect to do housework, such as repairing electrical appliances or mowing the lawn. But he or she has paid a lot in taking care of the children’s study, and I am satisfied on the whole.” to describe in more detail. This may make the subjects’ subjective feelings more realistic and the results may be more accurate. It is not due to the differences in coding of researchers that lead to the significance of the results.

Previous studies have confirmed that theoretically there is a strong two-way causal relationship between marital satisfaction and depression, and there is gender difference in the causal relationship between husband and wife. It tests the longitudinal data between husband and wife through cross-lagged stability model, recursive model and nonrecursive model [49]. Marital satisfaction has a slight potential impact on depression, and the impact on wife is more significant. For husbands, early depression has a potential impact on later marital satisfaction. A study of Iranian [50] pregnant women showed that marital life satisfaction, spouse education level and work income were related to the reduction of depression symptoms of pregnant women. Emma nagy et al. [51] reported that socio-economic factors played a role in influencing the risk of depression in women. This paper only analyzes the correlation between marital satisfaction and depression. Through previous studies, it can be seen that the two may affect each other. The information of depression is mixed in marital satisfaction, and there is a simultaneous bias. In this case, marital satisfaction is correlated with the error term, which violates the hypothesis of the classical ordinary least square (OLS), which is one of the conditions that cause endogeneity. In future research, it is good to look for an Instrumental Variables (IV) that is highly related to the explanatory variables but not to the random error term. Use IV and the regression coefficient in the model to obtain a consistent estimator, and use econometric methods to explore this endogenous problem. However, a better study design to explain causality is a longitudinal study design.

Other limitations of this article are as follows. Firstly, the study used cross-sectional data, which can only be used to compare the influence of different numbers of children on the life satisfaction and depression of couples in other families and cannot offer insights into the dynamic changes in couples’ mental health when new children are born in the family. Secondly, only three items of marriage satisfaction scale were used in this study. The follow-up study should adopt a more comprehensive marriage quality scale, which will help to further explore more specific areas of marriage quality that affect the psychological health of couples (i.e., trust, communication and sources of marital conflict).

## Conclusions

Based on descriptive statistics, wives experience more severe depression than husbands, and more detailed investigation should be carried out on the extent and causes of women’s marital dissatisfaction. In terms of measures to prevent depression, women’s mental health should be given more priority than men’s. Although the willingness of couples to have children in China and other low-fertility countries is currently low, and some couples are also worried about the burdens (e.g., economic burden) of raising a new born. This study found that living in a large family with more children is beneficial for couples’ mental health, and the increase of the husband’s marital satisfaction score will more effectively reduce the wife’s depression score. Accordingly, couples may do well to have more children and live with their relatives to form large families. Meanwhile, in the process of preventing depression among couples, mental health workers should not only emphasize the promotion of marital satisfaction but also consider whether the husband or wife has a neurotic personality, and carry out special treatment and preventive measures to promote the mental health of couples more effectively. Notably, this study found that it is especially important to work with neurotic personalities in wives. These findings highlight the importance of considering dyadic dynamics in marital relationships to better understand the factors affecting couples’ mental health and formulate appropriate prevention strategies.

## Data Availability

The data analyzed in this study were released to the researchers without access to any personal data. The dataset supporting the conclusions of this article is available in the CFPS repository, http://www.isss.pku.edu.cn/cfps/.

## Abbreviations

APIM: Actor–Partner Interdependence Model
APIMoM: Actor–Partner Interdependence Moderation Model
CFPS: China Family Panel Studies
MDMD: Marital Discord Model of Depression
MLM: Multilevel mode
OLS: Ordinary least square
IV: Instrumental variables
